# Accuracy of the national violent death reporting system in identifying unintentional firearm deaths to children by children

**DOI:** 10.1186/s40621-024-00499-0

**Published:** 2024-07-02

**Authors:** Samuel Fischer, Matthew Miller, Catherine Barber, Deborah Azrael

**Affiliations:** 1 Harvard Injury Control Research Center, Harvard T. H. Chan School of Public Health, Boston, MA USA; 2 Department of Health Sciences, Northeastern University, 360 Huntington Avenue, Boston, MA 02115-5000 USA

**Keywords:** Firearm, Pediatric, Mortality, Unintentional, Injury, Violence, Surveillance

## Abstract

**Background:**

In assigning manner of death (MOD) for inclusion on death certificates, medical examiners and coroners do not always apply uniform criteria. Previous research indicates surveillance statistics based on death certificates, such as the National Vital Statistics System, grossly miscount unintentional firearm deaths. The National Violent Death Reporting System (NVDRS) has taken steps to reduce variability in manner of death coding by providing uniform criteria for assigning an “abstractor manner of death” (AMD). AMD has five categories: unintentional, suicide, homicide, undetermined, and legal intervention homicide. A previous study found good accuracy of AMD coding for unintentional firearm deaths, all ages, 2003–2006, but a more recent study reported that the NVDRS undercounted self- and other-inflicted unintentional firearm deaths in which both the victim and shooter (for other-inflicted injuries) were under age 15 (2009–2018).

**Findings:**

We replicated the recent study’s sample population, identifying 924 NVDRS incidents from 2009 to 2018 in which both victim and, for other-inflicted injuries, shooter age was under 15 and AMD was homicide, suicide, unintentional or undetermined (there were no legal intervention deaths to children). We assigned a researcher-adjudicated MOD (RMD) by reviewing incident narratives. RMD was compared with AMD and with manner recorded on the death certificate. Based on RMD as the gold standard, the sensitivity, specificity, and predictive values positive and negative of the AMD for unintentional childhood firearm deaths were, respectively, 90%, 99%, 98% and 96%; 86% (24/28) of false negatives were coded by abstractors as homicides. By contrast, death certificate manner had relatively poor sensitivity (63%).

**Conclusions:**

In our sample of 924 deaths, the abstractor manner of death generally agreed with researcher-adjudicated manner of death, though not perfectly, missing 10% of researcher-adjudicated unintentional deaths, mostly because abstractors coded these unintentional deaths as homicides. A sizable minority of false negatives were unintentional deaths where the narrative explicitly noted that adult negligence contributed to a child’s unintentional shooting death. While AMD coding in NVDRS is good, it could be improved if NVDRS coding guidelines explicitly affirmed that potential prosecution for negligent manslaughter is not a contraindication to an AMD of unintentional, provided the firearm was not used to intentionally harm, threaten, or coerce.

## Introduction

National Vital Statistics System (NVSS) data grossly miscount unintentional firearm injuries, undercounting cases among children and overcounting cases among adults (Barber, 2002; Barber and Hemenway 2011). One reason for these inaccuracies is that when assigning manner of death (MOD) for inclusion on death certificates, coroners and medical examiners do not always apply uniform criteria. (Centers for Disease Control and Prevention 2022; Hanzlick et al. 2015).

An alternative source of data on violent deaths, the National Violent Death Reporting System (NVDRS), takes steps to reduce variability in MOD coding by providing uniform criteria for assigning an “abstractor manner of death” (AMD) (Centers for Disease Control and Prevention 2022). Rules instruct abstractors, for example, to code fatalities from Russian roulette as suicides, regardless of MOD assigned by coroners and medical examiners.

A previous study focusing on early years in the NVDRS, 2003–2006, found that AMD coding for unintentional firearm deaths was accurate (Barber and Hemenway 2011), but a more recent study, published in this journal, reported that the NVDRS greatly undercounted self- and other-inflicted unintentional firearm deaths in which both the victim and (for other-inflicted injuries) the shooter were under age 15 (Vaishnav et al. 2023). In the latter study, the authors reported that between 2009 and 2018 the NVDRS correctly coded only 61% of the unintentional firearm deaths identified by the researchers (Vaishnav et al. 2023). The reason for this discrepancy and the types of cases miscoded is not clear. One possibility we attempted to assess is that the AMD is inaccurate. The current study adopts Vaishnav et al.’s case definition (and study period) to assess the accuracy of AMD relative to the manner determined by our research team after reviewing NVDRS narratives, 2009–2018. We also characterize discordant cases.

## Methods

We identified all firearm deaths that occurred among the 39 states that participated in NVDRS at any point between 2009 and 2018 in which victim age was under 15 years of age, and if other-inflicted, the suspect’s age was determined to be under age 15. After reviewing incident narratives, we assigned a researcher-adjudicated MOD (RMD). RMD was compared with AMD and with the manner recorded on the death certificate. Specificity, sensitivity, and predictive values positive (PVP) and negative (PVN) were calculated for the AMD and for death certificate manner based on RMD as the gold standard.

## Results

We identified 924 firearm deaths in NVDRS of which 273 were adjudicated by our team as unintentional. Comparing AMD and RMD, the sensitivity, specificity, PVP and PVN of the AMD for unintentional firearm deaths were, respectively, 90%, 99%, 98% and 96% (Table 1). 86% (24/28) of false negatives were coded by abstractors as homicides. At least 25% of the false negatives pertained to cases where the narrative explicitly noted adult negligence (e.g., negligent manslaughter) while also describing an unintentional shooting by a child, or described an unintentional shooting by a child being prosecuted for manslaughter or homicide (not shown). Comparing RMD and death certificate MOD, the sensitivity, specificity, PVP and PVN were, respectively, 63%, 99.9%, 99%, and 86%. By contrast, death certificate manner had relatively poor sensitivity (of 63%).

**Table 1:** Accuracy of manner of death for unintentional firearm deaths to children by children, NVDRS, 2009–2018

		Gold standard (researcher-adjudicated MOD)				Totals
		Unintentional	Homicide	Undetermined	Suicide	
	Gold Standard Total	273	40	16	595	924
Abstrator Manner	Unintentional	245	0	2	2	249
	Homicide	24	40	0	0	64
	Undetermined	4	0	14	1	19
	Suicide	0	0	0	592	592
DC Manner	Unintentional	171	0	0	1	172
	Homicide	84	39	0	1	124
	Undetermined	10	0	13	3	26
	Suicide	4	0	0	586	590
	Pending Investigation	3	1	3	3	10
	Missing Data	1	0	0	1	2

## Discussion

In our sample of 924 deaths, the AMD had high levels of agreement with our researcher-adjudicated manner of death. However, NVDRS abstractors were not perfect, missing 10% of cases our research team identified as unintentional firearm deaths, mostly because abstractors coded unintentional firearm deaths as homicides. While AMD coding in NVDRS is good, it could be improved if NVDRS coding guidelines explicitly affirmed that AMD should be coded as unintentional when the shooter did not intend harm and was not using the firearm to threaten or coerce another person. This includes cases where the shooter or the adult owner of the gun might be held criminally liable for negligent manslaughter, as might be the case if an adolescent unintentionally shoots another during horseplay with a parent’s gun that they assumed was unloaded. This recommendation could be applied to victims of all ages as the logic underlying it directs the abstracter to focus on the shooter’s intent, not on legal definitions of liability.

## Data Availability

The dataset analyzed during the current study is available by request via the NVDRS Restricted Access Database application process: https://www.cdc.gov/violenceprevention/datasources/nvdrs/dataaccess.html.

## Abbreviations

AMD: Abstractor Manner of Death
CME: Coroner / Medical Examiner
DC: Death Certificate
MOD: Manner of Death
NVDRS: The National Violent Death Reporting System
PVP: Positive Predictive Value
PVN: Negative Predictive Value
RMD: Researcher–adjudicated Manner of Death
